# Novel therapeutic approach: organic arsenical (melarsoprol) alone or with *all-trans* -retinoic acid markedly inhibit growth of human breast and prostate cancer cells in vitro and in vivo

**DOI:** 10.1054/bjoc.1999.0942

**Published:** 2000-01-18

**Authors:** K Koshiuka, E Elstner, E Williamson, J W Said, Y Tada, H P Koeffler

**Affiliations:** Division of Hematology/Oncology, Department of Medicine, Department of Pathology, UCLA School of Medicine, Cedars-Sinai Medical Center, 8700 Beverly Blvd, LA, CA 90048, USA; Second Department of Surgery, Yamanashi Medical University, Yamanashi, Japan

**Keywords:** melarsoprol, retinoid, breast cancer, prostate cancer, apoptosis

## Abstract

The organic arsenical known as melarsoprol (Mel-B) is used to treat African trypanosomiasis. Recently, another arsenical, As_2_O_3_was shown to be effective in treatment of acute promyelocytic leukaemia. We have investigated the anti-tumour activities of Mel-B either with or without *all-trans* -retinoic acid (ATRA) using the MCF-7 human breast cancer cells, as well as the PC-3 and DU 145 human prostate cancer cells both in vitro and in vivo. The antiproliferative effects of Mel-B and/or ATRA against breast and prostate cancer were tested in vitro using clonogenic assays and in vivo in triple immunodeficient mice. Furthermore, the mechanism of action of these compounds was studied by examining the cell cycle, levels of bcl-2, apoptosis and antiproliferative potency using a pulse-exposure assay. Clonogenic assays showed that the cancer cell lines were sensitive to the inhibitory effect of Mel-B (effective dose that inhibited 50% clonal growth [ED_50_]: 7 × 10^−9^M for MCF-7, 2 × 10^−7^M for PC-3, 3 × 10^−7^M for DU145 cells. Remarkably, the combination of Mel-B and ATRA had an enhanced antiproliferative activity against all three cancer cell lines. Furthermore, the combination of Mel-B and ATRA induced a high level of apoptosis in all three cell lines. Treatment of PC-3 and MCF-7 tumours growing in triple immunodeficient mice with Mel-B and ATRA either alone or in combination markedly retarded tumour size and weight of the tumours without major side-effects. In conclusion, our results suggest that either Mel-B alone or with ATRA may be a useful, novel therapy for breast and prostate cancers. © 2000 Cancer Research Campaign


*British Journal of Cancer* (2000) **82**(2), 452–458
© 2000 Cancer Research Campaign
Article no. bjoc.1999.0942

### Novel therapeutic approach: organic arsenical (melarsoprol) alone or with *all-trans*-retinoic acid markedly inhibit growth of human breast and prostate cancer cells in vitro and in vivo

**K Koshiuka[1], E Elstner[1], E Williamson[1], JW, Said[2], Y Tada[1] and HP Koeffler[1]**

[1]Division of Hematology/Oncology, Department of Medicine and [2]Department of Pathology, UCLA School of Medicine, Cedars-Sinai Medical Center, 8700 Beverly Blvd, LA, CA 90048, USA; Second Department of Surgery, Yamanashi Medical University, Yamanashi, Japan

**Summary** The organic arsenical known as melarsoprol (Mel-B) is used to treat African trypanosomiasis. Recently, another arsenical, $As_2O_3$ was shown to be effective in treatment of acute promyelocytic leukaemia. We have investigated the anti-tumour activities of Mel-B either with or without *all-trans*-retinoic acid (ATRA) using the MCF-7 human breast cancer cells, as well as the PC-3 and DU 145 human prostate cancer cells both in vitro and in vivo. The antiproliferative effects of Mel-B and/or ATRA against breast and prostate cancer were tested in vitro using clonogenic assays and in vivo in triple immunodeficient mice. Furthermore, the mechanism of action of these compounds was studied by examining the cell cycle, levels of bcl-2, apoptosis and antiproliferative potency using a pulse-exposure assay. Clonogenic assays showed that the cancer cell lines were sensitive to the inhibitory effect of Mel-B (effective dose that inhibited 50% clonal growth [$ED_{50}$]: $7 \times 10^{-9}$ M for MCF-7, $2 \times 10^{-7}$ M for PC-3, $3 \times 10^{-7}$ M for DU145 cells. Remarkably, the combination of Mel-B and ATRA had an enhanced antiproliferative activity against all three cancer cell lines. Furthermore, the combination of Mel-B and ATRA induced a high level of apoptosis in all three cell lines. Treatment of PC-3 and MCF-7 tumours growing in triple immunodeficient mice with Mel-B and ATRA either alone or in combination markedly retarded tumour size and weight of the tumours without major side-effects. In conclusion, our results suggest that either Mel-B alone or with ATRA may be a useful, novel therapy for breast and prostate cancers. © 2000 Cancer Research Campaign

**Keywords:** melarsoprol; retinoid; breast cancer; prostate cancer; apoptosis

Breast and prostate cancers are the most common malignant diseases among women and men, respectively in Europe and the USA (Harris et al, 1997). Surgical resection or radiation therapy are potentially curative for localized diseases. Advanced breast and prostate cancers are associated with a poor prognosis, and conventional chemotherapies and radiation therapy are still of limited effectiveness. Endocrine therapies usually lead to either a partial or complete remission. However, subsequent relapse often occurs, and the disease re-emerges within a few years. Innovative approaches for advanced disease are necessary.

Arsenic is a naturally occurring element; pure arsenic is not common in the environment. Rather, it is usually found combined with one or more other elements such as oxygen, chlorine and sulphur. Arsenic combined with these elements is referred to as inorganic arsenic, whereas arsenic combined with carbon and hydrogen is referred to as organic arsenic. Maintaining a distinction between inorganic and organic arsenic is important, since the organic forms are usually less toxic. Traditional Chinese medicine has used an arsenic-containing remedy (Ai-Lin1) for many different ailments (Mervis, 1996). In the 1940s and 1950s, Fowler's solution which contained 1% potassium arsenite was frequently used for the treatment of chronic myelogenous leukaemia (Donofrio et al, 1987). Administration of inorganic arsenic, arsenic trioxide ($As_2O_3$), produced a high complete remission rate, as well as a relatively long-term survival in a significant proportion of individuals with acute promyelocytic leukaemia (APL) (Sun et al, 1992; Zhang et al, 1996; Chen et al, 1997; Soignet et al, 1998). Melarsoprol (Mel-B), an organic arsenical synthesized by complexing melarsen oxide with dimercaprol, has primarily been used for the treatment of African trypanosomiasis (Apted, 1970; Milord et al, 1992; van Nieuwenhove, 1992; Yunmbam et al, 1993). Mel-B induced apoptosis and inhibited in vitro growth of B-cell chronic lymphocytic leukaemia cell lines (König et al, 1997).

Retinoïds are natural and synthetic derivatives of vitamin A (Bollag et al, 1992). They prevent development as well as growth of several tumour types in animal models; and clinical trials have shown efficacy in individuals with APL, leukoplakia and recurrent squamous tumours of the head and neck (Gudas, 1992). Furthermore, retinoids inhibit the in vitro growth of a variety of cancer cells including those from leukaemias, breast, prostate and pancreas cancers (Douer et al, 1981; Pienta et al, 1993; Teelmann et al, 1993; Bollag et al, 1994; de Vos et al, 1996; Elstner et al, 1996). The regulation of cell growth and differentiation of normal, premalignant, and malignant cells by retinoids results from their effects on gene expression. These effects are mediated by nuclear retinoid receptors, which are ligand-activated transcription factors and members of the steroid hormone receptor superfamily (Evans, 1988; Mangelsdorf et al, 1994). Action of retinoids is mediated either by retinoic acid receptor-α (RAR-α), RAR-β, RAR-γ,

*Accepted 10 February 1999*
*Revised 22 July 1999*
*Received23 July 1999*

*Correspondence to:* HP Koeffler and/or by retinoic X receptors (RXR) (Petkovich, 1992). *All-trans*-retinoic acid (ATRA) is the first highly effective differentiation-inducing agent for remission induction in patients with APL (Huang et al, 1988; Warrell et al, 1993). It is the ligand for RARs.

To our knowledge, this is the first report of the effects of Mel-B and the combination of Mel-B with ATRA for solid tumours. We found that Mel-B or the combination of Mel-B and ATRA had potent anti-tumour activity against human breast and prostate cancer cells both in vitro and in vivo.

### MATERIALS AND METHODS

### Mice

Twenty male and twenty female 8-week-old BNX *nu/nu* mice were purchased from Harlan Sprague Dawley Inc, (Indianapolis, IN, USA) and were maintained in pathogen-free conditions with irradiated chow.

### Cell culture

The human DU 145 and PC-3 prostate cancer cell lines and the MCF-7 breast cancer cell line were obtained from American Type Culture Collection (Rockville, MD, USA) and were maintained in RPMI-1640 medium (Gibco Laboratories, Grand Island, NY, USA) supplemented with 10% fetal calf serum (FCS; Gibco), 100 U $ml^{-1}$ penicillin and 100 mg $ml^{-1}$ streptomycin.

### Drugs

Melarsoprol {p-[(4,6,-diamino-s-triazin-2-yl)amino]dithiobenzene-arsonous acid 3-hydroxypropylene ester} was a gift from the Central Disease Control (Atlanta, GA, USA), prepared in 180 mg/5 ml ampoules and stored at 4°C. ATRA (Sigma, St Louis, MO, USA) was dissolved in dimethyl sulphoxide (DMSO) at $10^{-2}$ M for in vitro studies and 50 mg $ml^{-1}$ for in vivo studies. It was stored at –80°C and protected from light.

### Clonogenic assay in soft agar

Potency of drugs to inhibit the clonogenic growth of cancer cells and the resulting ED50s (effective dose which inhibited 50% of clonal growth) was determined by extensive dose–response studies in soft agar. The cells from 60–80% confluent liquid cultures were plated into 24-well flat bottom plates using a two-layer, soft-agar system with a total volume of 400 µl, as described previously (Munker et al, 1986). Drugs were added on day 0 prior to addition of feeder layer to the culture plates. After 14 days of cultivation, colonies (> 50 cells) were counted with an inverted microscope. All experiments were done independently at least three times in triplicate dishes per experimental point.

### Western blot analysis of bcl-2

MCF-7, PC-3 and DU 145 cells were seeded at $1 \times 10^5$ and allowed to adhere overnight. The medium was replaced and to it was added either Mel-B ($10^{-6}$ M) and/or ATRA ($10^{-7}$ M). The cells were incubated with these compounds for 72 h. Lysates were prepared using Triton X-100 lysis buffer (20 mM Tris. Cl pH 8.0, 137 mM NaCl, 10% glycerol, 1% Triton X-100, 2 mM EDTA, and 1 mM sodium orthovanadate). One hundred micrograms of the protein extract was added to each lane of a 10–20% gradient poly-acrylamide gel. The membrane was blocked in phosphate-buffered saline (PBS)/5% non-fat milk (MLK) at room temperature and subsequently incubated with murine monoclonal anti-Bcl-2 (clone100, Santa Cruz Biotechnology, Santa Cruz, CA, USA) at 1 mg $ml^{-1}$ in PBS/3% MLK for 2 h at room temperature, followed by incubation with horseradish peroxidase conjugated anti-mouse Ig (Amersham, Arlington Heights, IL, USA) at 1:1500 in PBS/3% MLK. An actin monoclonal antibody from Oncogene Research Products was used as a control protein. The results were visualized by enhanced chemiluminescence (Amersham), and densitometry was performed using the Alpha Imager 2000 digital imaging system with Alpha Ease version 3.0 software (Alpha Innotech Corp., San Leandro, CA, USA).

### Cell cycle analyses

Cell cycle was analysed by flow cytometry. Briefly, cancer cells at <60% confluency were cultured either with or without analogues for 3 days in tissue culture medium, trypsinized, washed in DPBS, fixed in methanol and incubated for 30 min at 4°C in the dark with a solution of 5 mg $ml^{-1}$ propidium iodide, 1 mg $ml^{-1}$ RNAase (Sigma), and 0.1% Nonidet P-40 (Sigma). Analysis was performed immediately after staining using the CELLFit program (Becton Dickinson) whereby the S phase was calculated with a RFit model.

### Measurement of apoptosis

After 5 days of cultivation of cancer cells with or without drugs (Mel-B, $10^{-6}$ M; ATRA, $10^{-7}$ M), the cells were trypsinized, washed with DPBS and analysed for apoptosis. Activation of an endo-nuclease results in extensive DNA cleavage and, thus, generates a large number of DNA strand breaks in apoptotic cells. DNA frag-mentation was confirmed in our study by labelling of DNA strand breaks in apoptotic cells with BrdUTP (Li et al, 1995). This deoxynucleotide, once incorporated into the DNA strand breaks, is detected by a fluorescein isothiocyanate (FITC)-conjugated anti-BrdUrd antibody (Becton Dickinson, San Jose, CA, USA). Morphologically, cells undergoing apoptosis possess prominent features such as intense staining, highly condensed and/or frag-mented nuclear chromatin, a general decrease in overall cell size, and cellular fragmentation into apoptotic bodies. These features make apoptotic cells relatively easy to distinguish from necrotic cells. For morphology, cytospin-slides with cultured cells were stained by Diff-Quick Stain Set. Apoptotic cells were enumerated in a total of about 300 cells by light microscopy.

### Animal treatment protocol

Several animal studies were performed, using male BNX mice for the PC-3 prostate cancer experiments and female BNX mice for the MCF-7 breast cancer cells. Animals were bilaterally, subcuta-neously injected with $5 \times 10^6$ of either PC-3 or MCF-7 cells per tumour in 100 µl Matrigel (Collaborative Biomedical Products, Bedford, MA, USA). Before injection of cells, the animals received 300 rads whole body irradiation. The mice were divided into four groups of five mice each, and received either diluant (DMSO) or experimental agents. Mel-B and ATRA (50 µl per injection) were administered intraperitoneally thrice weekly. One day after tumour

© 2000 Cancer Research Campaign injections, mice were treated with either Mel-B alone, ATRA alone, or the combination of Mel-B and ATRA. Doses of Mel-B and ATRA were chosen from our preliminary studies in which various doses thrice weekly were given and mice were followed for toxicity. During the experiments, two mice died: one receiving Mel-B (10 mg $kg^{-1}$) and the other in the ATRA group (7.5 mg $kg^{-1}$) cohort. The cause of their deaths was unknown. Tumours were measured every week with vernier calipers. Tumour size was calculated by the formula: a × b × c, where a is the length and b is the width and c is the height in millimetres. At the end of the experiments, blood was collected from the orbital sinus for serum chemistries and haematopoietic analyses using Dupont Analyst Benchtop Chemistry System (Dade International, Newark, DE, USA) and by Serono-Baker 9000 Diff (Biochem Immuno-Systems, Allentown, PA, USA) respectively. Animals were sacrificed by carbon dioxide asphyxiation and tumour weights were measured after their careful resection.

### Histology

Tumours and normal organs from sacrificed mice were fixed in 10% neutral buffered formalin and embedded in paraffin wax prior to histologic sectioning. Sections were stained with haematoxylin and eosin, and tumour necrosis and fibrosis were evaluated. Controls consisted of tumours and organs from mice not subjected to treatment.

### Statistical analysis

All numerical data were expressed as the average of the values obtained, and standard deviation (s.d.) was calculated. For the in vitro studies, significance was determined by conducting a paired Student's *t*-test. For the in vivo studies, the statistical significance of the difference was analysed by the non-parametric Mann–Whitney *U*-test.

### RESULTS

### In vitro studies

#### *Clonogenic assay*

To study the effects of Mel-B, ATRA or their combination on clonogenic growth of cancer cells, the two-layer soft-agar system was performed. The MCF-7 breast cancer cells were very sensitive to the inhibitory effect of Mel-B in clonogenic assay ($ED_{50}$: $7 \times 10^{-9}$ M); the prostate cancer cell lines, PC-3 and DU 145 had $ED_{50}$s of $2 \times 10^{-7}$ M, and $3 \times 10^{-7}$ M respectively (Figure 1). The ATRA alone at $10^{-7}$ M was only moderately inhibitory of the clonogenic growth of the PC-3 or DU 145 prostate cancer cells (30% and 27% respectively); however, the same concentration of ATRA ($10^{-7}$ M) inhibited about 70% clonal growth of the MCF-7 breast cancer cells (Figure 2). Interestingly, the combination of both drugs (Mel-B, $10^{-7}$ M) and ARTA, $10^{-7}$ M) had at least an additive effect on clonal inhibition of each of the cancer cell lines (PC-3: 65% inhibition; DU 145: 74% inhibition; MCF-7: 90% inhibition) (Figure 2).

### Apoptosis and bcl-2 levels

Exposure of the cells to the combination of Mel-B ($2 \times 10^{-6}$ M) and ATRA ($10^{-7}$ M) for 5 days synergistically induced apoptosis in each of the cell lines [DU 145 (42%), PC-3 (51%) and MCF-7 cells (59%)], as measured by DNA fragmentation as compared to exposure of the cells to either agent alone (Figure 3). A similar, dramatic effect was observed when assessing apoptosis by morphology (data not shown).

The effect of Mel-B ($10^{-6}$ M, 5 days), ATRA ($10^{-7}$ M, 5 days) or

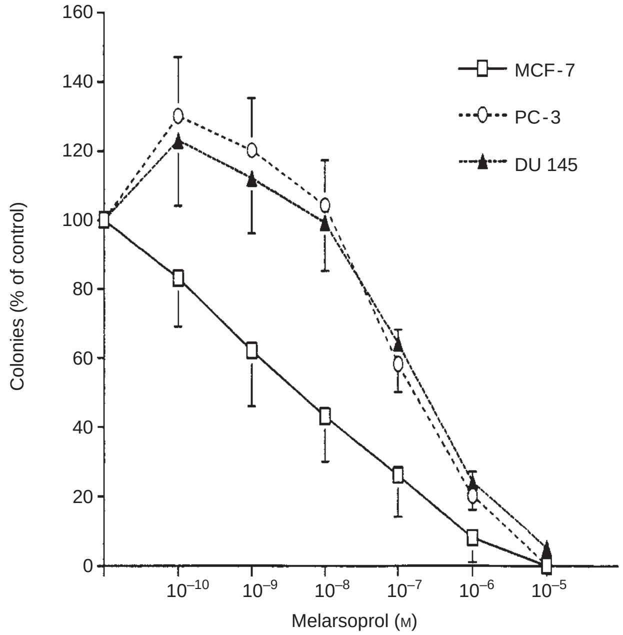

**Figure 1** Dose–response studies of Mel-B: effect on clonal proliferation of breast and prostate cancer cells. Results are expressed as a mean per cent ± s.d. of control plates containing no drug. Each point represents a mean of at least three experiments with each experimental point having triplicate dishes. The drug was added to the culture dishes on day 0

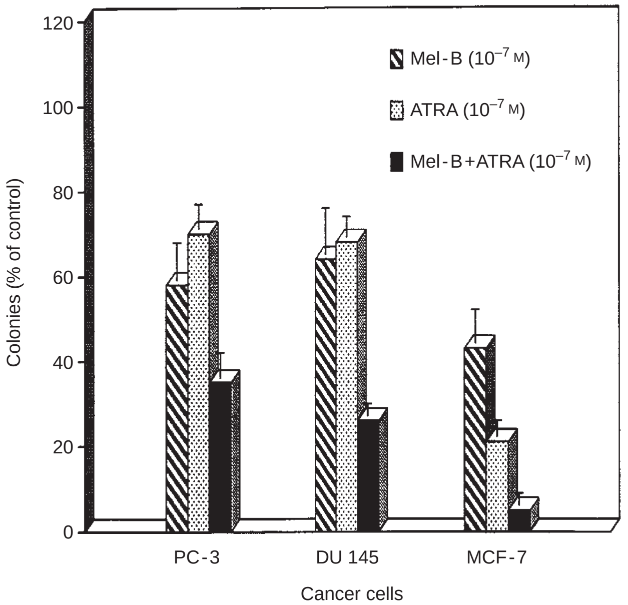

**Figure 2** Effect of Mel-B and/or ATRA on clonal proliferation of breast and prostate cancer cells. Results are expressed as a mean percent ± s.d. of control plates containing no drug. Each point represents a mean of at least three experiments with each experimental point having triplicate dishes. The compounds were added to the culture dishes on day 0

© 2000 Cancer Research Campaign

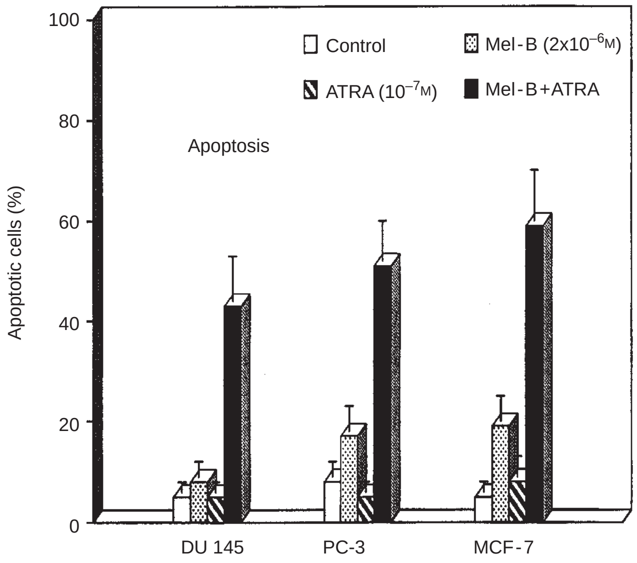

**Figure 3** Apoptosis of cancer cells, as measured by labelling of DNA strand breaks with BrdUTR, after exposure of cells for 5 days to either Mel-B ($2 \times 10^{-6}$ M), ATRA ($10^{-7}$ M) or both. Data expressed as per cent of apoptotic cells and represent the mean ± s.d. of two experiments. Controls are untreated cancer cells

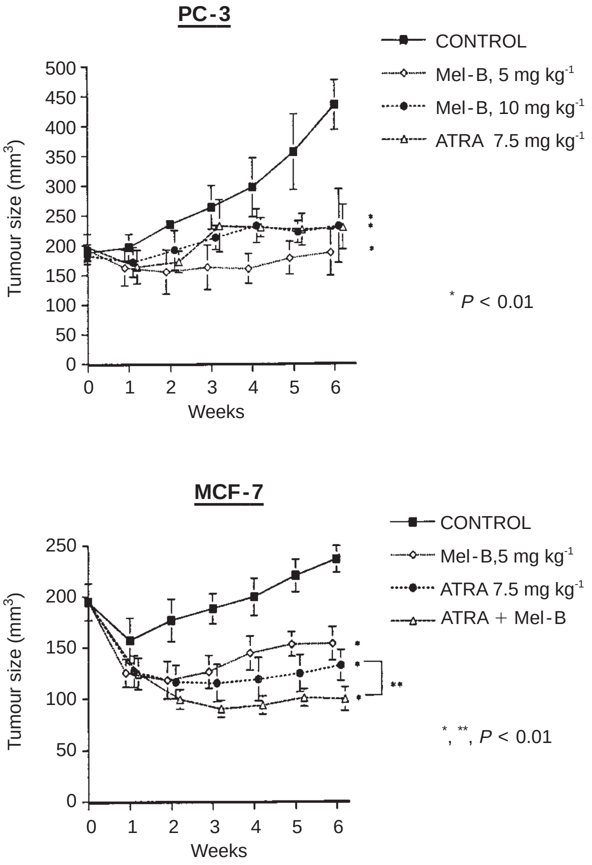

**Figure 4** Volume of MCF-7 and PC-3 tumours in BNX mice receiving Mel-B and ATRA. Human MCF-7 breast or PC-3 prostate cancer cells ($5 \times 10^6$) were injected subcutaneously, and Mel-B and ATRA were administered intraperitoneally (M, W, F), for 6 weeks to the BNX nude mice. Tumour volumes were calculated as the product of the length, width and height (see Materials and Methods section) of each tumour. Data are expressed as the mean ± s.d. for eight to ten tumours. Definitions: *, significantly different from control groups with $P < 0.01$ and **, significantly ($P < 0.01$) different between ATRA (7.5 mg $kg^{-1}$), and Mel-B and ATRA groups as determined by Mann–Whitney *U*-test both on the cellular content of bcl-2 protein was measured by Western blot (data not shown). When bcl-2 expression was corrected for actin expression, Mel-B decreased levels by 13%, ATRA lowered expression by 73%, and both together caused an 86% decrease in bcl-2 levels in the MCF-7 cells. Levels of bcl-2 were lower in the untreated PC-3 and DU 145 cells as compared to MCF-7, and these levels did not change markedly after exposure to Mel-B and/or ATRA (data not shown).

### In vivo studies

We tested the ability of Mel-B and/or ATRA to inhibit the growth of MCF-7 breast and PC-3 prostate cancer cells growing in triple immunodeficient mice. Figure 4 shows the effect of Mel-B and/or ATRA on the size of PC-3 and MCF-7 tumours during 6 weeks of therapy. All of the treatment groups had statistically significantly smaller tumours than the diluant-control groups. Administration of Mel-B and ATRA alone remarkably suppressed the growth of the tumours. The size of the PC-3 tumours in mice treated with Mel-B at 5 and 10 mg $kg^{-1}$ was similar, although the former was slightly smaller. The most potent effect was observed when Mel-B and ATRA were administered together to the mice bearing MCF-7 tumours. Besides determining the volume of the tumours over time, they were carefully dissected at the termination of the study and weighed. Results paralleled the volume measurements (data not shown). Tumour weights from each of the treated groups were statistically different from those of the control group, and the combination of Mel-B and ATRA was more potent than either alone. During the study, all mice were weighed once per week. The body weights of all treated groups were 91–101% of that of the control groups (data not shown). In general, all the mice of each of the cohorts looked healthy.

The blood chemistries (ten different studies including BUN, creatinine, liver enzymes and electrolytes) and haematopoietic parameters (including peripheral blood white and red cell counts, platelet count and white cell differential evaluation) were obtained at the end of the study. The blood was collected from the orbital sinus while the animals were anaesthetized. No difference in the mean values of the blood chemistries were observed between the treated and untreated animals (data provided on request). The blood haematopoietic data showed little change between the cohorts, Group H (Mel-B + ATRA) had slightly higher white blood cell and platelet counts (data provided on request).

### Histology

### *MCF-7*

Tumours from control mice revealed poorly differentiated adenocarcinomas with about 30% necrosis and fibrosis (Figure 5A). Tumours from mice treated with Mel-B showed increased necrosis and apoptotic bodies (about 60% of the tumour mass) as well as fibrosis (Figure 5B). Tumours from mice receiving ATRA alone revealed similar changes in about 40% of each tumour section (data not shown). Tumours from mice treated with the combination of ATRA and Mel-B also contained a similar amount of tumour necrosis and apoptosis (Figure 5C).

© 2000 Cancer Research Campaign

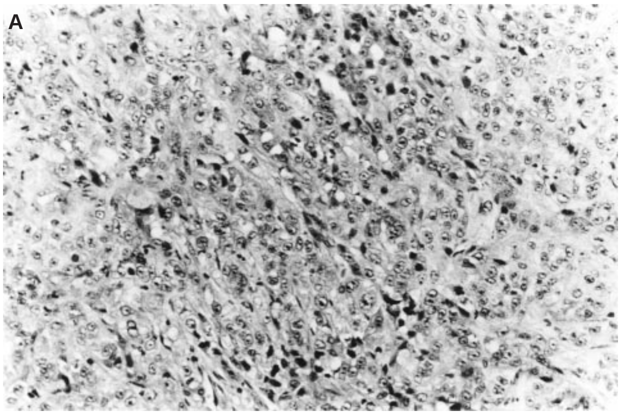

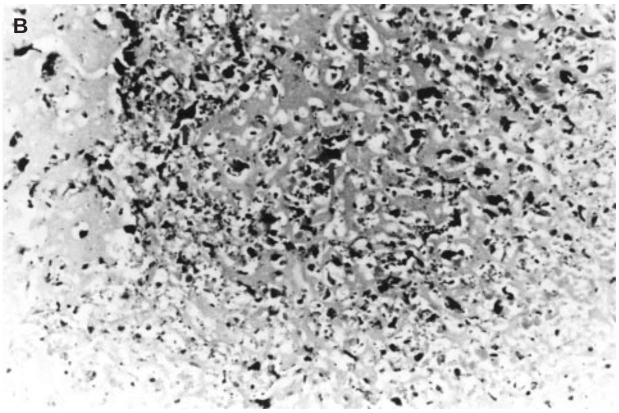

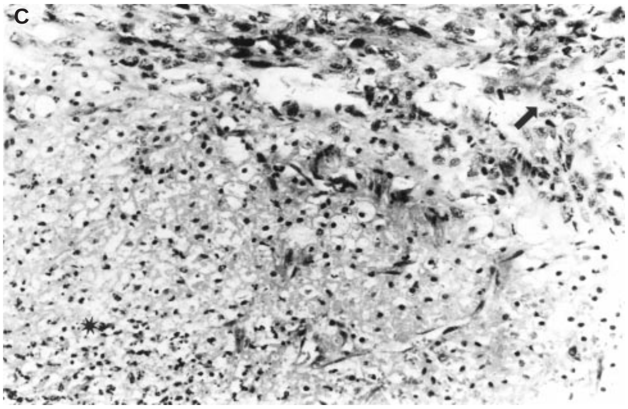

**Figure 5** Histological findings of MCF-7 breast tumours at the end of treatment. (**A**) MCF-7 tumour from control mice demonstrating infiltrating poorly differentiated adenocarcinoma (see arrow). The malignant cells appear in sheets with large nuclei, prominent nucleoli, and abundant cytoplasm. There is no evidence of tumour necrosis. (**B**) MCF-7 tumour harvested from mice treated with Mel-B, showing extensive necrosis and apoptosis (see arrow). There is scant viable tumour remaining at the top of the field (arrow). The remaining tumour shows only outlines of necrotic cells with pyknotic nuclei. Dense apoptotic bodies are present (asterisk). (**C** MCF-7 tumour obtained from mice treated with Mel-B plus ATRA, displaying extensive necrosis and no viable tumour. Dense aggregates of chromatin (arrows) are present in a background of acellular tissue necrosis

### *PC-3*

Controls revealed poorly differentiated carcinomas with small foci of necrosis and fibrosis which constituted approximately 20% of the area of the tumour section (data not shown). Tumours from mice treated with Mel-B at 5 or 10 mg $kg^{-1}$ and/or ATRA 7.5 mg $kg^{-1}$ revealed extensive necrosis and fibrosis [50–60% of each of the tumour sections revealed necrosis and histologic changes of apoptosis including formation of apoptotic bodies, and fibrosis involved approximately 30% of the tumour area (data not shown)].

### DISCUSSION

Arsenic compounds have been generally considered to be a poison and a potent environmental carcinogen for human skin and lung cancers (Jaafar et al, 1993; Dong et al, 1994). Nevertheless, the inorganic arsenical, $As_2O_3$, induced apoptosis in the NB4 APL cell line associated with decreased expression of bcl-2 mRNA and protein (Chen et al, 1996, 1997). Furthermore, clinical studies in China and the USA have shown that $As_2O_3$ is an effective drug for patients with APL (Sun et al, 1992; Zhang et al, 1996; Chen et al, 1997; Soignet et al, 1998). Laboratory data suggest that the activity of arsenic in haematopoietic cell lines was independent of the expression of PML-RARα fusion product which is specific for the APL cells (König et al, 1997). The organic arsenical, Mel-B, is used for treatment of trypanosomiasis and this arsenical has been formulated for human use since 1949 (Friedheim, 1949). Recently, Mel-B was found to inhibit growth and decrease expression of bcl-2 in several chronic B-cell leukaemia cell lines (JVM-2, 183CLL, WSU-CLL) (König et al, 1997). Because arsenicals may have a broad range of activity, we examined the ability of Mel-B to inhibit the growth of human breast and prostate cancer cell lines.

Our clonogenic growth assays showed that MCF-7 breast cancer cells were very sensitive to the inhibitory activity of Mel-B. Furthermore, the strongest combined effects were observed in the cells treated with Mel-B and ATRA (Figures 1 and 2). The DU 145 prostate cancer cells are notably resistant to a variety of in vitro therapies (Thompson, 1994; Israel et al, 1995; deVos et al, 1996; Campbell et al, 1998); therefore, the sensitivity of these cells to clonal inhibition of proliferation by Mel-B and their prominent apoptosis with the combination of Mel-B and ATRA is notable. The PC-3 prostate cancer cells are moderately resistant to a variety of agents (Thompson, 1994; Israel et al, 1995; deVos et al, 1996; Campbell et al, 1998). These cells were also inhibited in their clonal growth by Mel-B. The Mel-B and ATRA at least additively decreased clonal growth of these prostate cancer cells. Comparison of the potency of inorganic $As_2O_3$ to Mel-B showed that Mel-B was about tenfold more potent in its antiproliferative activities than was $As_2O_3$ for each of the breast and prostate cancer cell lines, suggesting that Mel-B may be more active than $As_2O_3$ (data not shown).

The mechanism by which Mel-B mediates its anticancer activity is unclear. Prior investigation have reported that arsenicals affect protein tyrosine phosphorylation (Cavigelli et al, 1996; Chen et al, 1998). They can also decrease levels of glutathione which can result in DNA damage as a result of increased intracellular reactive oxygen molecules (Snow, 1992; Cavigelli et al, 1996).

 *© 2000 Cancer Research Campaign*

Apoptosis or programmed cell death is of importance for the development and homeostasis of multicellular organisms (Fisher, 1994). Specific therapies have been designed to enhance the susceptibility of human cancers to undergo apoptosis (Yonish-Rouach et al, 1991; Symonds et al, 1994). Apoptosis is an active gene-directed cellular suicide mechanism; and many human genes contribute to its regulation, such as p53, c-myc and bcl-2 (Shi et al, 1992; Miyashita et al, 1993; Borsellino et al, 1995). We showed that the combination of Mel-B and ATRA dramatically and significantly increased the number of apoptotic cells in each of the three cancer cell lines, especially MCF-7 breast cancer cells (Figure 4). This effect was associated with a decrease in levels of bcl-2 protein in the MCF-7 cells (Figure 5). In contrast, levels of bcl-2 protein did not decrease in PC-3 and DU 145 prostate cancer cells after a similar treatment showing that decrease in existing levels of bcl-2 are not required for induction of apoptosis of these cells; but of note, bcl-2 levels were already relatively low in wild-type PC-3 and DU 145 cells. Soignet et al (1998) showed that apoptosis of APL cells by $As_2O_3$ was coincident with activation of caspases which are cysteine proteases important in mediating programmed cell death. Of interest after completion of our study, another investigative group noted that growth of $As_2O_3$-resistant NB4 APL cells was inhibited by the addition of ATRA to the cells (Gianni et al, 1998). Taken together, an arsenical and ATRA can have an enhanced antitumour effect in vitro; albeit, we do not understand the mechanism by which this occurs.

Our in vivo studies showed that the Mel-B as well as ATRA significantly inhibited the growth of PC-3 and MCF-7 cells. Furthermore, the treatment of MCF-7 breast cancer cells with the combination of Mel-B and ATRA was statistically superior to either Mel-B or ATRA alone. These data are consistent with our in vitro data. Blood chemistries and haematopoietic analyses showed that WBC and platelet numbers in the combination group were slightly higher than that of the control group, but all data were within the normal range (data available on request). Body weights in the experimental animals were within 10% of the control animals.

The histological data showed that all the MCF-7 breast and the PC-3 prostate tumours were poorly differentiated adenocarcinomas. Sections from mice treated with Mel-B and/or ATRA revealed extensive necrosis, apoptosis and fibrosis involving approximately 30–60% of the tumour area. Therefore, Mel-B had anticancer activities in vivo similar to what we observed in vitro. This activity occurred without major side-effects. The mechanisms of this anticancer effect remain unclear but are associated with prominent apoptosis. Toxicity was not discernible raising hopes that either Mel-B alone or when combined with ATRA may become a useful adjuvant therapy for breast and prostate cancers. This may be particularly true for the individuals who have minimal residual disease after curative attempt by surgery and/or radiotherapy.

### ACKNOWLEDGEMENTS

We thank Kim Burgin for her excellent secretarial assistance. This work was supported by NIH and US Defense grants, Parker Hughes Trust, C and H Koeffler Fund, CaP CURE Foundation and Aaron Eschman Trust. Dr Koeffler is a member of the UCLA Jonsson Comprehensive Cancer Center and holds an endowed Mark Goodson Chair of Oncology Research at Cedars-Sinai Medical Center, UCLA School of Medicine.

### REFERENCES

Apted FIC (1970) Treatment of human trypanosomiasis. In: *The African Trypanosomiases*, Mulligan HW (ed), pp. 684–710. George Allen & Unwin: London

Bollag W and Holdener EE (1992) Retinoids in cancer prevention and therapy. *Ann Oncol* **3**: 513–526

Bollag W and Peck R (1994) Cancer chemotherapy by combination of retinoids with cytokines and vitamin D analogs. Experimental and clinical results. *Ann Oncol* **5**: 17–22

Borsellino N, Belldegrun A and Bonavida B (1995) Endogenous interleukin 6 is a resistance factor for cis-diamminedichloroplatinum and etoposide-mediated cytotoxicity of human prostate carcinoma cell lines. *Cancer Res* **55**: 4633–4639

Campbell MJ, et al. (1998*a*) Expression of RARβ sensitizes prostate cancer cells to growth inhibition mediated by combinations of retinoids and a 19-nor hexafluoride D3 analog. *Endocrinology* **139**: 1972–1980

Campbell MJ, Dawson M and Koeffler HP (1998*b*) Growth inhibition of DU-145 prostate cancer cells by a Bcl-2 antisense oligonucleotide is enhanced by N-(2-hydroxyphenyl) all-trans retinamide. *Br J Cancer* **77**: 739–744

Cavigelli M, et al. (1996) The tumour promoter arsenite stimulates AP-1 activity by inhibiting a JNK phosphatase. *EMBO J* **15**: 6269–6279

Chen GQ, et al. (1996) In vitro studies on cellular and molecular mechanisms of arsenic trioxide ($As_2O_3$) in the treatment of acute promyelocytic leukemia: $As_2O_3$ induces NB4 cell apoptosis with downregulation of Bcl-2 expression and modulation of PML-RAR alpha/PML protein. *Blood* **88**: 1052–1061

Chen GQ, et al. (1997) Use of arsenic trioxide ($As_2O_3$) in the treatment of acute promyelocytic leukemia (APL): I. $As_2O_3$ exerts dose-dependent dual effects on APL cells. *Blood* **89**: 3345–3353

Chen W, et al. (1998) Tumour promoter arsenic activates extracellular signal-regulated kinase through a signaling pathway mediated by epidermal growth factor receptor and Sch. *Mol Cell Biol* **18**: 5178–5188

de Vos S, et al. (1996) Effects of retinoid X receptor (RXR)-class selective ligands on proliferation of prostate cancer cells. *Prostate* **32**: 115–121

Dong JT and Luo XM (1994) Effects of arsenic on DNA damage and repair in human fetal lung fibroblasts. *Mutat Res* **315**: 11–15

Donofrio PD, et al. (1987) Acute arsenic intoxication presenting as Guillain–Barré-like syndrome. *Muscle Nerve* **10**: 1114–1120

Douer D and Koeffler HP (1981) Retinoic acid: inhibition of the clonal growth of human myeloid leukemia cells. *J Clin Invest* **69**: 277–283

Elstner E, et al. (1996) Synergistic decrease of clonal proliferation, induction of differentiation and apoptosis of acute promyelocytic leukemia cells after combined treatment with novel 20-epi vitamin $D_3$ analogs and 9-cis retinoic acid. *J Clin Invest* **99**: 349–360

Evans RM (1988) The steroid and thyroid receptor superfamily. *Science* **240**: 889–895

Fisher DE (1994) Apoptosis in cancer therapy: crossing the threshold. *Cell* **78**: 539–542

Friedheim EAH (1949) The active form of tryparsamide TPB in the treatment of human trypanosomiasis. *Am J Trop Med* **29**: 173–180

Gianni M, et al. (1998) Combined arsenic and retinoic acid treatment enhances differentiation and apoptosis in arsenic-resistant NB4 cells. *Blood* **91**: 4300–4310

Gudas LJ (1992) Retinoids, retinoid-responsive genes, cell-differentiation, and cancer. *Cell Growth Diff* **4**: 655–662

Harris JR, Morrow M and Norton L (1997) In: *Cancer: Principles and Practice of Oncology*, DeVita VT, Hellman S and Rosenberg ST (eds), pp. 1557–1617. Lippincott-Raven: Philadelphia

Huang ME, et al. (1988) Use of *all-trans* retinoic acid in the treatment of acute promyelocytic leukemia. *Blood* **72**: 567–572

Israel K, Sanders BG and Kline K (1995) RRR-alpha-tocopherol succinate inhibits the proliferation of human prostatic tumor cells with defective cell cycle/differentiation pathways. *Nutrition Cancer* **24**: 161–169

Jaafar R, et al. (1993) Skin cancer caused by chronic arsenical poisoning – a report of three cases. *Med J Malaysia* **48**: 86–92

König A, et al. (1997) Comparative activity of melarsoprol and arsenic trioxide in chronic B-cell leukemia lines. *Blood* **90**: 562–570

Li X and Darzynkiewicz Z (1995) Labeling DNA strand breaks with BrdUTR. Detection of apoptosis and cell proliferation. *Cell Prolif* **28**: 571–579

Mangelsdorf DJ, Umesono K and Evans RM (1994) The retinoid receptors. In: *The Retinoids*, Sporn MB, Roberts AB and Goodman DS (eds), pp. 319–349. Raven Press: New York

Mervis J (1996) Ancient remedy performs new tricks [news]. *Science* **273**: 578

*© 2000 Cancer Research Campaign* 

Milord F, et al. (1992) Efficacy and toxicity of eflornithine for the treatment of *Trypanosoma brucei gambiense* sleeping sickness. *Lancet* **340**: 652–655
Miyashita T and Reed JC (1993) Bcl-2 oncoprotein blocks chemotherapy-induced apoptosis in a human leukemia cell line. *Blood* **81**: 151–157
Munker R, Norman AW and Koeffler HP (1986) Vitamin D compounds: effects on clonal proliferation and differentiation of human myeloid cells. *J Clin Invest* **87**: 424–430
Petkovich M (1992) Regulation of gene expression by vitamin A: the role of nuclear retinoic acid receptors. *Annu Rev Nutr* **12**: 443–471
Pienta KJ, Nguyen NY and Lehr JE (1993) Treatment of prostate cancer in the rat with the synthetic retinoid fenretinide. *Cancer Res* **53**: 224–226
Shi Y, Glynn JM and Guilgert LJ (1992) Role for c-myc in activation-induced apoptotic cell death in T cell hybridomas. *Science* **257**: 212–214
Snow ET (1992) Metal carcinogenesis: mechanistic implications. *Pharmacol Ther* **53**: 31–65
Soignet SL, Maslak P and Wang Z-G (1998) Complete remission after treatment of acute promyelocytic leukemia with arsenic trioxide. *New Engl J Med* **339**: 1341–1348
Sun HD, Ma L, Hu XC, et al. (1992) Arsenic trioxide treated 32 cases of acute promyelocytic leukemia. Chin *J Integrat Chin West Med* **12**: 170–171
Symonds H, et al. (1994) p53-dependent apoptosis suppresses tumor growth and progression in vivo. *Cell* **78**: 703–711
Teelmann K, et al. (1993) Comparison of the therapeutic effects of a new arytenoid, Ro 40-8757, and *all-trans*- and 13-cis-retinoic acids on rat breast cancer. *Cancer Res* **53**: 2319–2325
Thompson CB (1994) Apoptosis in the pathogenesis and treatment of disease. *Science* **267**: 1456–1462
van Nieuwenhove S (1992) Advances in sleeping sickness therapy. *Am Soc Belge Med Trop* **72**: 39–51
Warrell RP, Jr, et al. (1993) Acute promyelocytic leukemia. *N Engl J Med* **329**: 177–189
Yonish-Rouach E (1991) Wild-type p53 induces apoptosis of myeloid leukemic cells that is inhibited by interleukin-6. *Nature* **352**: 345–347
Yunmbam MK and Roberts JF (1993) In vivo evaluation of reuterin and its combinations with suramin, melarsoprol, DL-a-difluoromethyl-ornithine and bleomycin in mice infected with *Trypanosoma brucei*. *Comp Biochem Physiol* **105:**: 521–524
Zhang P, Wang SY and Hu XC (1996) Arsenic trioxide treated 72 cases of acute promyelocytic leukemia. *Chin J Hematol* **17**: 58–60

 *© 2000 Cancer Research Campaign*
